# *Pleurotus ostreatus*-Mediated Bioremediation of Polylactic Acid Microplastics: Unveiling a Sustainable Solution

**DOI:** 10.1021/acsomega.5c09999

**Published:** 2025-12-05

**Authors:** Christina N. Economou, Sine Mandrup Bertozzi, Giorgia Ferrari, Andrea Armirotti, Despina Fragouli, Athanassia Athanassiou

**Affiliations:** † Smart Materials, 121451 Istituto Italiano di Tecnologia (IIT), Via Morego 30, Genova 16163, Italy; ‡ Analytical Chemistry Facility, Istituto Italiano di Tecnologia (IIT), Via Morego 30, Genova 16163, Italy

## Abstract

Polylactic acid (PLA) is a compostable biopolymer with strong potential to replace petroleum-based plastics, yet its end-of-life degradation pathways under real-world conditions remain insufficiently understood at the molecular and structural levels. In this study, we investigate the physical–chemical mechanisms underlying the biodegradation of PLA microplastics by the edible white-rot fungus *Pleurotus ostreatus*. Detailed studies on the microplastics’ morphological, structural, and chemical changes reveal that after 30 days of fungal treatment, their surface presents small pores, with the concomitant formation of new carbonyl (C=O), carboxyl (−COOH), and hydroxyl (−OH) groups, consistent with oxidative scission of ester bonds. Concomitantly, differential scanning calorimetry and X-ray diffraction indicate a significant increase in crystallinity, attributed to a preferential enzymatic attack on amorphous domains, followed by chain reorganization. These observations elucidate the interplay between enzymatic oxidation (driven predominantly by laccase activity) and polymer chain mobility, demonstrating how enzymatic catalysis induces coupled chemical and morphological transformations in PLA. The findings provide a promising environmentally friendly solution for bioremediation of PLA materials by edible fungi, helping to overcome current limitations in industrial composting and enabling a standardized, efficient decomposition and a more predictable, sustainable end-of-life strategy within circular waste management systems. Furthermore, this approach offers new insights into the synthesis of lignocellulosic enzymes during the bioprocess.

## Graphical abstract

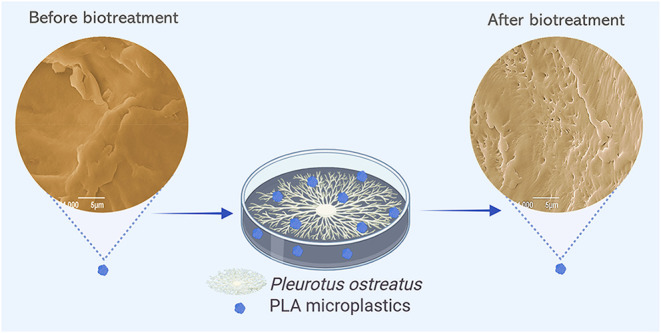

## 1 Introduction

Every year, over 400 million tons of plastics are produced worldwide,^1^ whereas it has been estimated that 19–23 million tons of plastic waste entered aquatic environments in 2016 and this figure is rising, arriving to between 20 and 53 million tons annually by 2030 if current trends continue.^2^ Since most plastics are not biodegradable, their environmental fate is governed by physical–chemical degradation pathways, like photo-oxidation, thermo-oxidation, and mechanical fragmentation, driven by solar radiation, oxygen, and mechanical stresses caused by winds or waves, resulting in their fragmentation into smaller particles. In particular, microplastics (MPs) (size: 5 μm–1 mm) and nanoplastics (size <1 μm) are found in natural habitats worldwide. Their slow chemical transformation alters their structure and surface, potentially affecting their bioavailability and human exposure.^3,4^

The plastic manufacturing industry is, therefore, moving to alternative materials for replacing fossil-based plastics with biobased and biodegradable polymers that are economically viable and environmentally friendly.^5^ Polylactic acid (PLA) is a thermoplastic aliphatic polyester of lactic acid derived from fermented biomass sources and is considered one of the most promising bioplastics for real-life applications. In fact, from the global bioplastic production, which is estimated to be 7.43 million tons in 2028, about 44% is destined for PLA production.^6^ PLA is mainly used for packaging applications due to its good mechanical and physical properties,^7^ while PLA-based microparticles and nanoparticles are already used in pharmaceuticals and cosmetics due to their high surface-to-volume ratio and the presumed low toxicity of lactic acid monomers in the human body.^8,9^ Regarding PLA’s biodegradability, it proceeds through hydrolytic and oxidative scission of ester linkages, but such transformations occur efficiently only under aerobic industrial composting conditions, at temperatures close to its glass transition temperature (Tg) (60–65 °C) and high relative humidity levels (∼60%).^10^

PLA biodegradation occurs in different stages since microorganisms cannot directly utilize the carbon contained in the polymer as an energy source due to its large molecular weight and low solubility in water. It involves the interfacial adsorption of microorganisms, followed by enzymatic depolymerization via ester bond cleavage, producing shorter oligomers and monomers. These chain scission events alter the molecular weight distribution, crystallinity, and surface energy, ultimately enabling microbial assimilation.^11–13^ PLA biodegradation depends on several properties of the material (e.g., molecular weight, degree of crystallinity) and on diverse environmental factors (e.g., pH, temperature, moisture, etc.).^14–16^ The shape and size of the PLA components also affect the biodegradation process, as particles with a larger surface area are more degradable than those with a smaller one due to increased contact between the microbes/enzymes and the polymer’s surface.^17^

Among different microbes, fungi are considered strong candidates for polymer degradation because their hyphae can penetrate the polymer matrix, increasing the surface area accessible to catalytic attack. Additionally, their oxidative and hydrolytic enzymes initiate chain scission, oxidation, and secondary structural rearrangements.^18^ In nature, white-rot fungi can efficiently break down lignin due to their complex ligninolytic enzyme systems comprising laccases and peroxidases, such as lignin peroxidases and manganese peroxidases, which play an important role in nutrient recycling.^19^ Such oxidative enzymes have strong potential to degrade synthetic polymers^20^ by generating reactive oxygen species (ROS), such as hydrogen peroxide (H_2_O_2_) and hydroxyl radicals (^•^OH)^21^ able to abstract hydrogen atoms from the polymeric backbone, creating carbon-centered radicals that react with oxygen and trigger further oxidative chain reactions in the polymeric chains.^22^

The white-rot fungus *P. ostreatus*, known as the oyster mushroom, is the most famous edible mushroom worldwide. For its growth, it utilizes lignocellulosic biomass^23^ and therefore it holds a promising role in bioremediation processes for agroindustrial residues and a broad range of chemical pollutants.^24–27^ *P. ostreatus* has also been used for the biodegradation of pretreated low-density polyethylene (LDPE) materials. After the 150 days of interaction with *P. ostreatus*, LDPE sheets demonstrated increased surface roughness and about a 30% increase for the Young′s modulus, while these effects were mainly attributed to the interaction of the polymer with the laccase, manganese peroxidase, and lignin peroxidase enzymes produced during the process.^28^ These findings were also confirmed by a recent study on the biodegradation of LDPE fragments with *P. ostreatus*, which proved an increased expression of laccase and manganese peroxidase genes during 90 days of treatment.^29^ When *P. ostreatus* was used to degrade common plastic bag strips mixed with paper towels, it was found that laccase, xylanase, and cellulase were the enzymes responsible for the creation of cracks and holes on the surface of the materials after 45 days of biotreatment.^30^ In another study, *P. ostreatus* was successfully used for the biodegradation of polyethylene plastic bag fragments previously exposed to sunlight for up to 120 days.^31^ Although the fungal biodegradation of polymers has received increasing attention, most studies provide only qualitative evidence of physicochemical changes, with limited mechanistic insight into the coupling between enzymatic oxidation, chain scission chemistry, and polymer morphological reorganization.^28,29,32–35^ Focusing on PLA, even though some studies have shown that several microbial enzymes such as lipase, esterase, protease, and cutinase, which can be found in fungi, bacteria, and algae,^13,33,36–39^ can effectively degrade this biopolymer through the hydrolytic route, to the best of our knowledge, studies on its enzymatic oxidation are scarce.^16^ Additionally, there is limited information on the fungal potential for the biodegradation of MPs,^40^ while no studies on the fungal degradation of PLA MPs have been conducted so far.

To this end, this study investigates the biodegradation of PLA MPs by the white-rot fungus *P. ostreatus*, with a focus on the physical–chemical mechanisms driving polymer breakdown. The synthesis of lignocellulolytic enzymes was monitored during the bioprocess to assess the fungus’s biochemical response under various MP loadings and to establish correlations between enzyme activity and specific chemical bond cleavage events in PLA. A comprehensive characterization of the polymer’s morphological, chemical, and structural evolution revealed that fungal oxidation preferentially targets amorphous domains, triggering ester bond scission, formation of oxygen-containing functional groups, and subsequent crystalline reorganization of the polymer matrix. These transformations were accompanied by a measurable consumption of lactic acid, confirming the metabolic assimilation of the degradation products. The findings demonstrate that *P. ostreatus* can mediate coupled chemical and structural modifications in PLA MPs at ambient temperature without pretreatment, offering a low-energy, sustainable degradation route grounded in fundamental polymer–enzyme interfacial chemistry.

## 2 Materials and Methods

### 2.1 PLA MPs, Fungal Strain, and Growth Conditions

All chemicals were purchased from Sigma-Aldrich for the preparation of culture media, enzymatic assays, and other analyses, and a Milli-Q Advantage A10 purification system was used to produce deionized water. Poly-L-lactic acid pellets (Ingeo 4043D, *M*_w_: 160 kDa, size: 4.00 mm) were purchased from Nature Works and transformed into MPs of sizes ranging between 0.30 and 0.75 mm through mechanical grinding using a dry mill (IKA, PILOTINA MC). The grinding procedure consisted of multiple stages, starting with the fragmentation of 4.00 mm PLA pellets using a 3.00 mm sieve. The ground material was then further processed using a finer 0.75 mm sieve to reduce the particle size. To isolate the desired size fraction (MPs ranging between 0.30 and 0.75 mm), the material was subjected to a final separation step using an electrical sieve (VWR test sieve 200 × 50 mm^2^) with a 0.30 mm mesh. Only the fraction retained between 0.30 and 0.75 mm was collected and used in the experimental work, as this size is commonly found in aquatic ecosystems.^41^

*P. ostreatus* DSM11191 was purchased from DSMZ (Germany) and maintained in potato dextrose agar (PDA, Merck). Experiments were performed in 100 mm Petri dishes containing 25 mL of a growth medium composed of the following: yeast extract (2.5 g/L); peptone (3.5 g/L); CaCO_3_ (0.1 g/L); and different concentrations of PLA MPs ranging from 7 to 80 g/L. In all experiments, the same cultivation conditions were kept, changing only the concentration of MPs to better evaluate the response of the fungus at PLA MPs. The liquid medium was autoclaved for 20 min at 120 °C (SYSTEC-VX 40) and PLA MPs were sterilized under ultraviolet (UV) light in a biohazard hood (VBH 48, ANGELANTONI LIFE SCIENCE Srl) for 1 h to avoid any microbial contamination during the experiments. The initial pH of the growth medium before and after sterilization was 7.2 ± 0.1 °C measured using a pH meter (Hanna Edge instrument). This initial pH value was chosen as a representative of aquatic ecosystems that exhibit a neutral pH, thereby aiming for more applicable results. Each Petri dish was inoculated with one agar plug of 8 mm cut from a 20 day-grown mycelium in a PDA Petri dish. Fungal cultures were incubated at 26 °C and 78% relative humidity, in the dark, which are ideal conditions for mycelial growth,^18,42^ for a period of one month in a climatic chamber (Memmert, HPP 260). Control experiments without the addition of MPs to evaluate the growth of the fungus in the growth medium and control experiments without the fungus to investigate the abiotic degradation of MPs at the same time (after 30 days) were also conducted at the same conditions used in each experiment. For all enzymatic assays, the fungal cultures were filtrated to separate the broth medium from mycelia and MPs using Whatman filters No. 1. The broth medium was then centrifuged at 5000 rpm for 10 min to obtain the crude enzymatic extract. For morphological, chemical, and crystallographic analyses after fungal treatment, a vigorous washing of MPs with distilled water was performed to remove mycelia followed by drying at 37 °C for 3 days. This temperature and drying time were applied to all PLA samples, including pristine PLA MPs (untreated MPs), to ensure that any change observed is attributed to the fungus treatment and not to thermal degradation or aging effects.

### 2.2 Analytical Methods

#### 2.2.1 Enzymatic Activity Assays

Laccase (EC 1.10.3.2) activity was measured using syringaldazine (4-hydroxy-3,5-dimethoxybenzaldehyde azine) as the substrate at 525 nm for approximately 10 min as previously described.^43^ One unit of laccase is the amount of enzyme that increases absorbance by 0.001 per minute under the specified assay conditions. Lignin peroxidase (E.C. 1.11.1.14) activity was determined based on the oxidation of the dye azure B.^44^ One unit of enzyme activity is defined as a 0.1 unit decrease in absorbance per minute per mL of the culture filtrate. Manganese peroxidase (E.C. 1.11.1.13) activity was determined by the oxidation of phenol red in the presence of hydrogen peroxide as previously described.^45^ All enzymatic activities were measured by a Cary 6000i UV–vis–NIR spectrophotometer (Agilent Technologies). For all enzymatic assays, the control consisted of the reaction medium with an inactivated crude enzymatic extract at 100 °C for 30 min.

#### 2.2.2 Scanning Electron Microscopy (SEM)

The surface morphology of dried PLA MPs (coated with 10 nm gold by sputtering) before and after fungal treatment was analyzed using a SEM JEOL JSM-6490LA microscope equipped with a secondary electron detector and applying a load current of 78 μA and a 10 kV acceleration voltage.

#### 2.2.3 Fourier Transform Infrared (FTIR) Spectroscopy

The chemical characteristics of dried MPs, both before and after fungal treatment, were analyzed using FTIR spectroscopy (VERTEX 70v, FTIR, Bruker) coupled with an attenuated total reflection (ATR) accessory (MIRacle ATR, PIKE Technologies). Spectral data were collected from 4000–600 cm^–1^ at a resolution of 2 cm^–1^ and 64 scans and the mean spectrum of four measurements (four different samples) is shown. All peaks were normalized to the reference peak at 1454 cm^–1^, corresponding to the asymmetric CH_3_ deformation modes (δ_as_CH_3_).^46,47^

#### 2.2.4 X-ray Diffraction (XRD)

XRD measurements of PLA MPs were conducted using a Malvern PANalytical Empyrean diffractometer equipped with a 1.8 kW Cu Kα ceramic X-ray tube and a PIXcel3D 2 × 2 area detector, operating at 45 kV and 40 mA. The degree of crystallinity was determined by dividing the crystalline area regions by the total area as previously described.^39^ All PLA samples were subjected to XRD analysis at the same time after biotreatment and subsequent drying to ensure similar aging effects.

#### 2.2.5 High-Performance Liquid Chromatography (HPLC)

Lactic acid, both its production during PLA MP biodegradation and its consumption by fungus, was analyzed and quantified using high-performance liquid chromatography with refractive index detection (HPLC-RID). An Agilent 1260 Infinity HPLC system equipped with a diode array detector (DAD) and a refractive index detector (RID) was used for these analyses. The analyses were performed on an Agilent Hi-Plex H PL1170–6830 column (300 × 7.7 mm ID, particle size 8 μM), using 5 mM H_2_SO_4_ in H_2_O as a mobile phase at 0.6 mL/min in isocratic flow. Prior to analysis, 1 M KOH (10 v/v%) was added to all calibrators, qualitative controls, and samples.

#### 2.2.6 Statistical Analysis

The experimental results are presented as the mean ± standard deviation (SD). Statistically significant differences between data were determined using one-way ANOVA analysis with 95% confidence intervals and Student’s *t* test (*t* test). The *p* value of ≤ 0.05 was considered statistically significant.

## 3 Results and Discussion

### 3.1 Fungal Growth on PLA MPs and Synthesis of Lignocellulosic Enzymes

Although fungal degradation of plastics has been widely reported in recent years,^18,29,34,35,48,49^ the molecular-level mechanisms and the role of specific microbial enzymes in driving polymer bond cleavage and structural rearrangements remain insufficiently understood. In this study, the white-rot fungus *P. ostreatus* was selected for biotreatment because of its ability to efficiently degrade polyesters, its high growth rate, and its exceptional capacity to produce laccase.^20,28,29,42^ To the best of our knowledge, there are currently no references in the literature detailing the application of laccase specifically for PLA degradation. Laccase is a copper-containing oxidase enzyme that facilitates the direct reduction of molecular oxygen (O_2_) to water (H_2_O), without hydrogen peroxide (H_2_O_2_) intermediate production, accompanied by the oxidation of an electron donor.^21,50^ Unlike lignin and manganese peroxidases, laccase does not require an external supply of H_2_O_2_, Mn^2+^, or other cofactors for oxidation, and it exhibits broad substrate specificity as well as stability over a wide range of pH and temperatures,^27,50,51^ properties that make it particularly effective in initiating radical-mediated oxidation and bond scission in polymer backbones.

Herein, *P. ostreatus* was cultivated in a defined growth medium, where the yeast extract and peptone served as nitrogen sources to facilitate the growth of mycelia. PLA MPs were added at concentrations of 7, 20, 40, and 80 mg/L to function as the primary carbon source, aiming to evaluate the fungus’s enzymatic response at different MP levels. As can be seen in Figure 1A,B, the surface of MPs used in this study was rough before fungal treatment, necessary for the facilitation of the cells’ attachment and growth.^52,53^ In general, fungal colonization of the polymer surface is a prerequisite for the induction of biodegradation because the formation of hyphal networks involves the secretion of small proteins called hydrophobins, which change the hydrophobicity of the surfaces, thus facilitating the attachment of filaments. During fungal colonization, extracellular polymeric compounds are also excreted to adhere mycelia tenaciously on the polymeric surface.^54,55^ In turn, the formation of the fungal biofilm takes place on the surface of the material, enhancing the interactions between microbes and the material surface. Subsequently, the production of enzymes starts utilizing the polymeric material as a nutrient source by breaking down the long-chain substances into smaller molecules which are then metabolized intracellularly through the tricarboxylic acid cycle.^40^ In our experiments, the colonization of PLA MPs by *P. ostreatus* after 15 days, when mycelia covered the surface of MPs, and the fungal biofilm formation after 30 days of cultivation are depicted in Figure 1C,D, respectively. This physical interaction indicates the possible assimilation of the PLA polymer chains as carbon sources for fungal growth. Note that in Figure 1, the washing protocol for MPs was not followed to demonstrate the physical interaction between the materials and the fungus.

**Figure 1:**
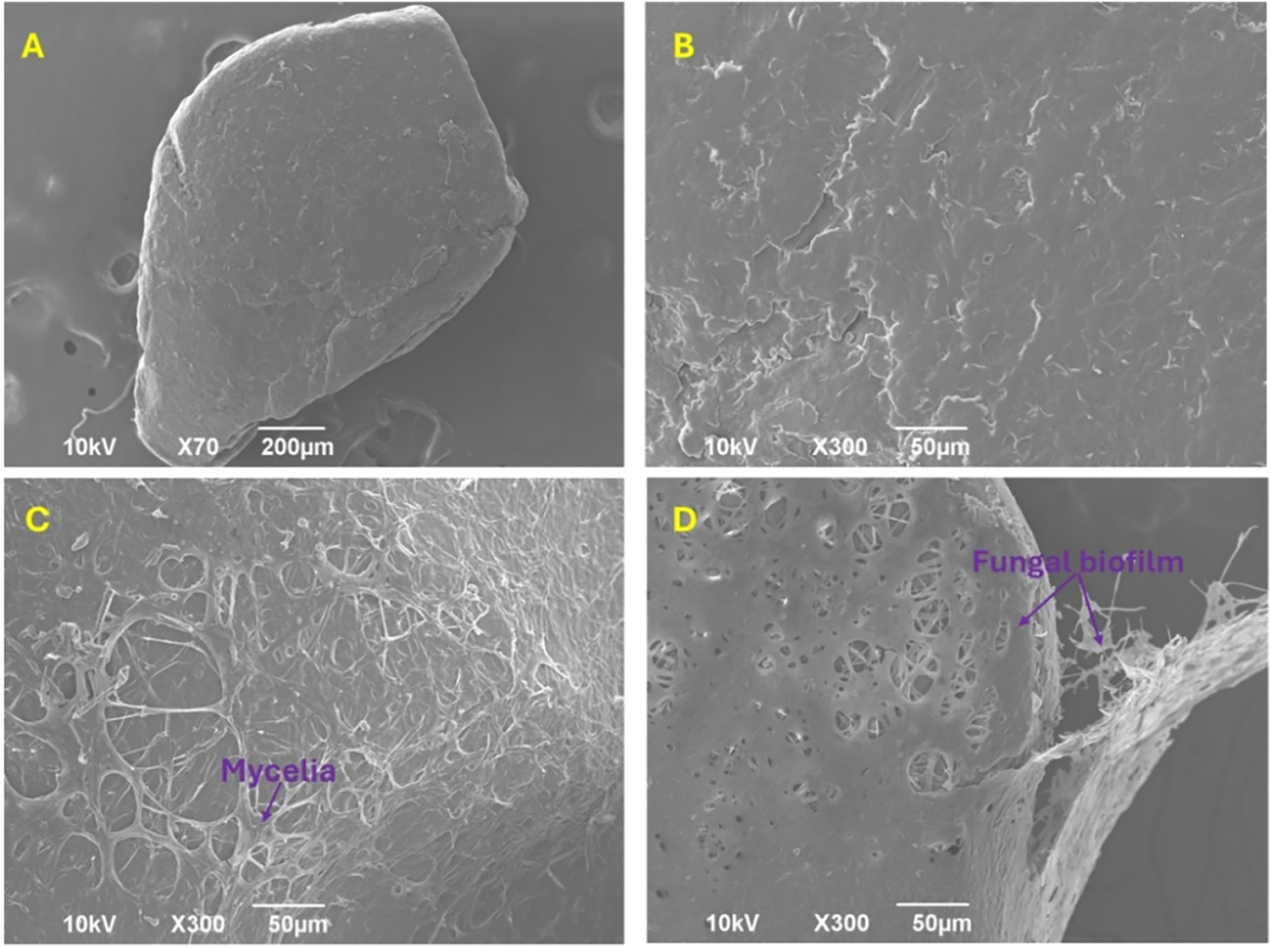
Representative SEM images showing the (A) shape and (B) surface of the untreated PLA MPs. (C) Colonized surface of PLA MPs by hyphal networks after 15 days and (D) fungal biofilm formation on the surface of PLA MPs after 30 days.

The major oxidative enzymes, i.e., laccase, lignin peroxidase, and manganese peroxidase, typically produced by white-rot fungi,^19^ were also quantified at intermediate and final stages of the fungal treatment of the MPs. This allowed us to correlate the temporal evolution of lignocellulolytic enzyme activity with the onset of oxidative reactions at the polymer surface, including radical formation, bond scission, and subsequent chemical and structural reorganization of the PLA matrix.

The profile of enzymes showed significant laccase production after 15 days of treatment, which almost doubled at the end of the treatment after 30 days for experiments with MP concentrations of 7–40 g/L (Table 1). For these experiments, the one-way ANOVA analysis revealed that laccase enzyme synthesis was not statistically significant after 15 days (F­(2,6) = 2.62, *p* = 0.15), but it was statistically significant after 30 days (F­(2,9) = 7.58, *p* = 0.01). In the experiments with the MP concentration of 80 g/L, laccase activity was almost 16-fold higher compared to the other experiments after 15 days of biotreatment (∼800 vs ∼50 U/mL), whereas a statistically nonsignificant reduction (*t* test, *p* = 0.26) of about 25% (∼600 U/mL) was observed after 30 days (Table 1). Additionally, manganese peroxidase and lignin peroxidase were not detected in the fungal cultures for all of the tested MP concentrations. Furthermore, it is worth mentioning that no enzymatic activity in the control experiments (without PLA MPs) was detected after 15 days, while negligible laccase activity (∼4 U/mL) was detected at the end of the fungal cultivation. Overall, it seems there is a positive correlation between laccase synthesis and the MP concentration, as laccase activity was increased with the increasing concentration, suggesting that the fungus can recognize PLA as a carbon source for its survival. Furthermore, an optimum carbon to nitrogen ratio seems to exist, given that the initial nitrogen concentration was the same for all experiments conducted, while the MP/carbon concentration was varied. In general, the carbon to nitrogen ratio is considered one of the key parameters for laccase production, using lignocellulosic substrates, whereas the optimum ratio varies between fungal strains and substrate sources.^42,56^ Moreover, the synthesis of fungal enzymes is affected by the physicochemical characteristics of the substrate and environmental factors such as temperature and pH.^57^ In the present study, the temperature during the experiments was kept in the optimal range for the growth of *Pleurotus* species (26 °C).^23^ The initial pH of culture media was neutral, while at the end of the biotreatment, the pH reached an increase of up to 8.0–8.3. Probably, in the present study, manganese peroxidase and lignin peroxidase were not detected because the optimum pH for their synthesis should be slightly acidic,^58,59^ while nonoptimal pH conditions may cause a decreased expression of ligninolytic peroxidase genes.^59^ Indeed, previous studies have shown that the types of polymer substrates and the treatment conditions significantly affect the type of enzymes produced by *P. ostreatus* during biodegradation. Specifically, laccase and manganese peroxidase were the major enzymes detected in cultures of *P. ostreatus* after 30 days of treatment of LDPE sheets at pH 7.0, while lignin peroxidase was produced after 60 days of biotreatment.^28^ Laccase was also detected after 45 days of treatment of plastic strips mixed with paper towels using the same fungus.^30^ On the contrary, laccase was not detected after 7 days of biotreatment of polystyrene and polyethylene by *P. ostreatus*, demonstrating that the fungus required a longer time to colonize and degrade such types of polymers.^60^

**Table 1:** Laccase Activity (U/mL) during *P. ostreatus* Biotreatment of PLA MPs^a^

experiment	15 days	30 days
control experiments (without PLA MPs)	n.d.^a^	3.74 ± 1.28^a^
7 g/L PLA MPs	34.17 ± 12.76^b^	64.74 ± 26.78^b^
20 g/L PLA MPs	53.60 ± 9.99^b^	110.35 ± 21.87^c^
40 g/L PLA MPs	45.97 ± 8.12^b^	80.71 ± 8.70^d^
80 g/L PLA MPs	800.75 ± 184.26^e^	620.36 ± 118.05^e^

a Data are presented as mean values of triplicate experiments ± standard deviation. n.d.: not detected. Different letters indicate significant differences between the mean enzyme activities of the samples (*p* ≤ 0.05).

### 3.2 Morphological, Chemical, and Crystallographic Modifications of PLA MPs

Figure 2 depicts the morphological changes on the surface of PLA MPs before and after 30 days of fungal treatment. As can be seen, contrary to the compact surface of the MPs before any treatment, the particles’ surface after the treatment was covered by submicrometric pores in all of the MP concentrations tested. These observations confirm the significant physical transformation at the MPs’ surface, indicative of enzymatic degradation. This is in accordance with previous studies which have shown that *P. ostreatus* can create cracks and holes on the surface of plastic strips^30^ and on LDPE fragments.^29^ Morphological changes on the surfaces of PLA sheets have also been observed after treatment with the fungus *Trichoderma viride* which produces lignocellulosic enzymes.^16^ In the present case, morphological modifications caused by the *P. ostreatus* treatment were observed at MPs when their concentration in the medium was between 20 and 80 g/L. The MPs, when their concentration was lower (i.e., 7 g/L), did not exhibit any morphological changes (Figure 1S) probably due to lower laccase enzyme production; thus, they were excluded from further analysis. Also, no modifications were observed in the abiotic control experiments conducted in the same growth medium (pH 7) in the absence of fungi (Figure 2S). These observations are in agreement with the literature that has demonstrated that moderate alkaline or neutral conditions do not affect the morphological and physicochemical characteristics of PLA even when higher temperatures (i.e., 37 °C) are applied for the same duration.^39^

**Figure 2:**
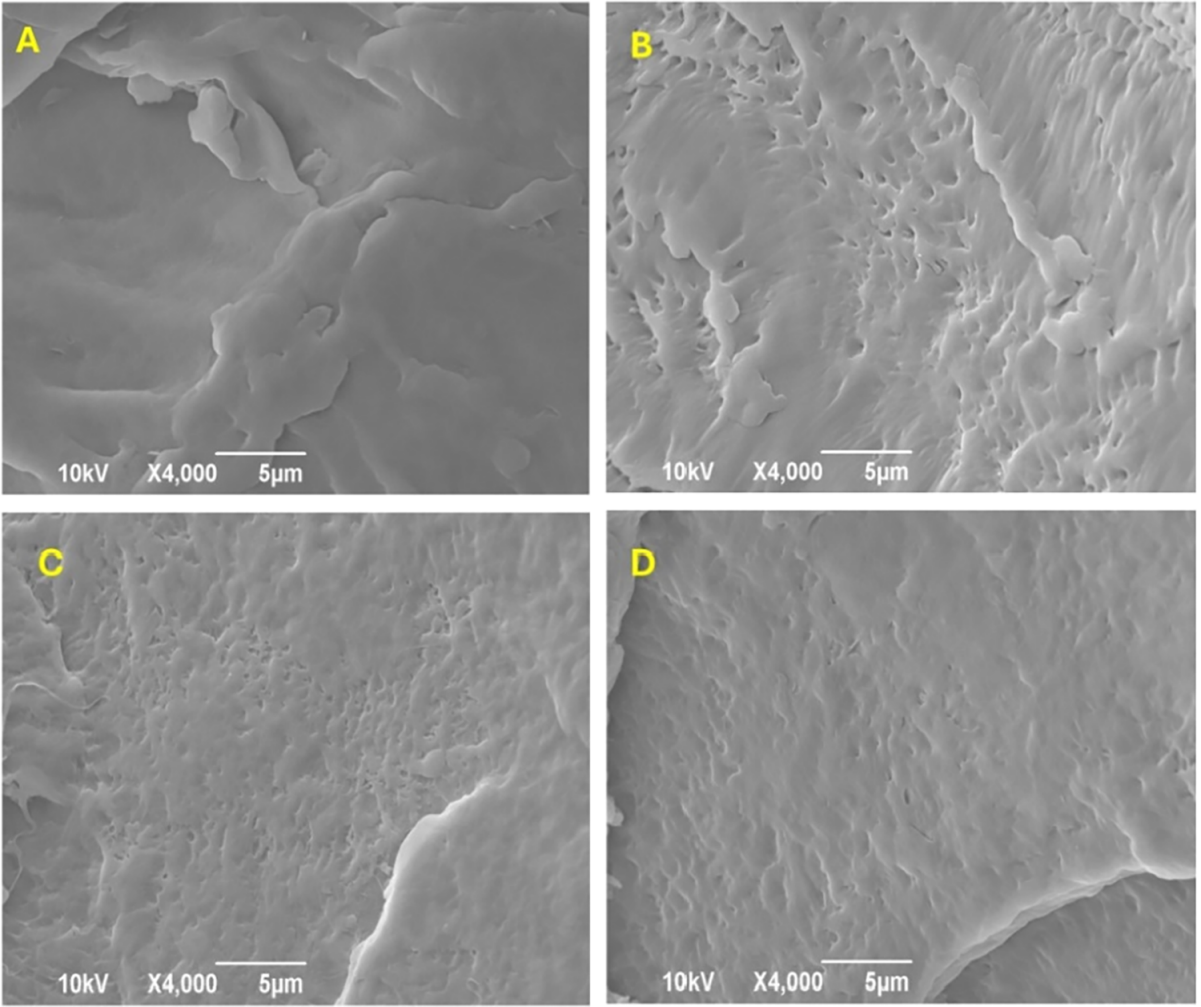
SEM images of the surface of PLA MPs (A) before and after 30 days of *P. ostreatus* biotreatment with PLA MP concentrations in the growth medium of (B) 20, (C) 40, and (D) 80 g/L.

FTIR analysis was also performed to investigate whether fungal activity, in addition to physical changes, could also cause chemical modifications in the MP structure. As shown in Figures 3a and 3Sa,b, untreated PLA showed typical absorption bands corresponding to the C=O stretching (*v*C=O) of lactic acid esters at 1746 cm^–1^, CH_3_ stretching at 1454 cm^–1^, and C–O–C stretching vibrations of ester groups at 1300–1000 cm^–1^.^46,61^ Additionally, the CH stretching bands of low intensities related to the methyl groups in PLA are observed at 2996 cm^–1^ (CH_3_ asymmetric stretching, ν_as_CH_3_), 2946 cm^–1^ (CH_3_ symmetric stretching, ν_s_CH_3_), 2920 cm^–1^ (CH_2_ asymmetric stretching, ν_as_CH_2_), 2882 cm^–1^ (C–H stretching, νCH), and 2850 cm^–1^ (CH_2_ symmetric stretching, ν_s_CH_2_).^61^ The peaks between 970 and 850 cm^–1^ are associated with the crystalline structure and helical conformation of PLA. The peak at 754 cm^–1^ is assigned to the δC=O in-plane bending and the peak at 705 cm^–1^ corresponds to the γC=O out-of-plane bending.^39^

**Figure 3:**
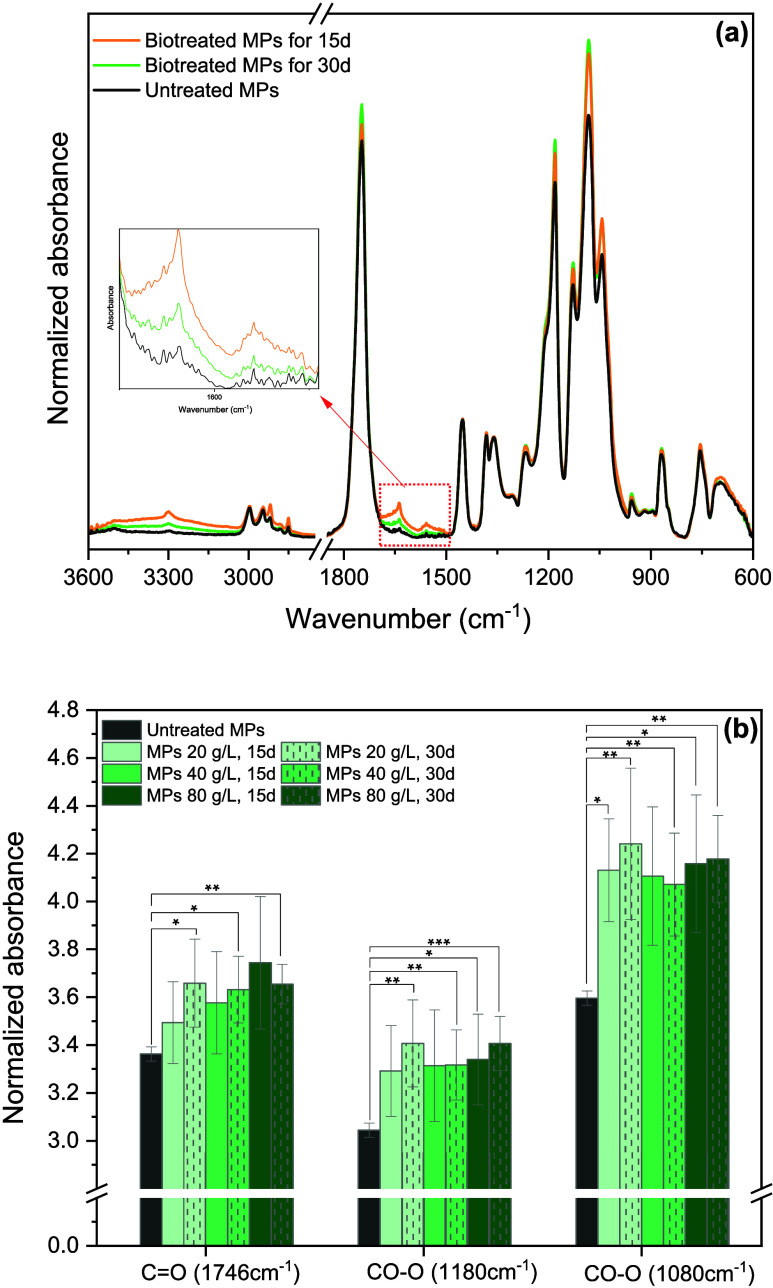
(a) Normalized ATR-FTIR spectra of PLA MPs of 20 g/L in the growth medium after 15 and 30 days of *P. ostreatus* biotreatment in comparison with the untreated MP spectrum. (b) Normalized absorbance for the peaks at 1746, 1180, and 1080 cm^–1^ of PLA MPs (20, 40, and 80 g/L) in the growth medium after 15 and 30 days of *P. ostreatus* biotreatment in comparison with the untreated MPs. The asymmetric bending of CH_3_ at 1454 cm^–1^ was used as the reference peak. Data represent the mean values of four independent measurements ± standard deviation. The statistical significance was determined by the *t* test comparing each group of biotreated MPs to the untreated ones (*p ≤ 0.05, **p ≤ 0.01, *** *p* ≤ 0.001).

In all cases, after the fungal treatment, an increased intensity in the carbonyl stretching (*v*C=O) at 1746 cm^–1^ and in the asymmetric and symmetric stretching vibration of the ester group (CO–O stretch) at 1180 and 1080 cm^–1^, respectively, was observed, probably due to the chain scission reaction associated with the formation of carbonyl and/or carboxyl groups caused by oxidation reactions.^47,62^ This increment can be better represented by the normalized absorbance values of these characteristic peaks at 1746, 1180, and 1080 cm^–1^ using the absorbance value at 1454 cm^–1^ assigned to the asymmetric bending of the CH_3_ group as a reference peak (Figure 3b). For the normalized absorbance of 1746 cm^–1^, a statistically significant increment (*t* test, *p* < 0.05) was observed after 30 days of biotreatment, while the normalized absorbance of 1180 cm^–1^ showed a statistically significant increase in most cases, with exceptions being experiments with MP concentrations of 20 and 40 g/L after 15 days of biotreatment. For the absorbance of 1080 cm^–1^, a statistically significant increment was noted for all experiments (*t* test, *p* < 0.05), except for those with an MP concentration of 40 g/L after 15 days of biotreatment. The degradation of MPs was also confirmed by the enhanced intensity of the peaks at 1640 and 1560 cm^–1^ (Figure 3a), indicating the formation of both acid and anionic forms of nonesterified carboxyl groups (COO−), respectively.^63,64^ Similar observations related to these two peaks have been reported in the literature during the biodegradation of PLA.^39,65^ Additionally, the appearance of a new peak at 3300 cm^–1^ after fungal treatment indicates the formation of hydroxyl (−OH) end groups.^47^ These results prove that the chemical structure of PLA MPs changed upon chain scission, with more oxygenic groups in the MP structure after fungal treatment due to the action of the laccase enzyme.^21^ The oxygenic groups can increase the hydrophilicity of PLA, affecting water absorption as well as its susceptibility to further degradation.^39^ Interestingly, the intensity of the peaks associated with carboxyl and hydroxyl groups (at 1640, 1560, and 3300 cm^–1^) significantly reduced after prolonged biotreatment (i.e., after 30 days). This might be because the fungus used the generated products as nutrient sources for its survival.^48^ It is to be noted that these chemical changes were not observed in control experiments (PLA MPs in the growth medium without the fungus), as the FTIR spectra of pristine PLA MPs (untreated MPs) were identical to the spectra of PLA MPs in the growth medium without the fungus (Figure 4S).

To evaluate possible structural changes after fungal treatment, the crystallinity of the PLA MPs was estimated. During biodegradation, the crystallinity of semicrystalline PLA is expected to increase because enzymes penetrate the amorphous phase of the polymer structure more easily than the crystalline one, leaving more resistant crystalline domains.^66^ In all cases studied, the MPs displayed the typical peaks at 2θ–16.7° (200/110 plane), 2θ–15.0° (010 plane), and 2θ–19.0° (203/113 plane), as well as some other weak peaks, all of which have been assigned to α-phase semicrystalline PLA based on the ICDD 00–064–1624 database (Figure 4). The degree of crystallinity of the untreated PLA MPs was estimated to be 28.86 ± 0.05% in agreement with the crystallinity range of PLA (0–37%).^67^ However, the crystallinity degree statistically increased (*p* < 0.05) up to 37.16 ± 0.03, 33.43 ± 0.05, and 34.47 ± 0.03% for MP concentrations of 20, 40, and 80 g/L, respectively, confirming the degradation of the amorphous PLA phase.^15,47^ This agrees with the FTIR results, which demonstrated the chain scission of PLA following fungal degradation, and with SEM images, which revealed morphological changes on the surface of the PLA MPs, all contributing to the increased crystallinity.

**Figure 4:**
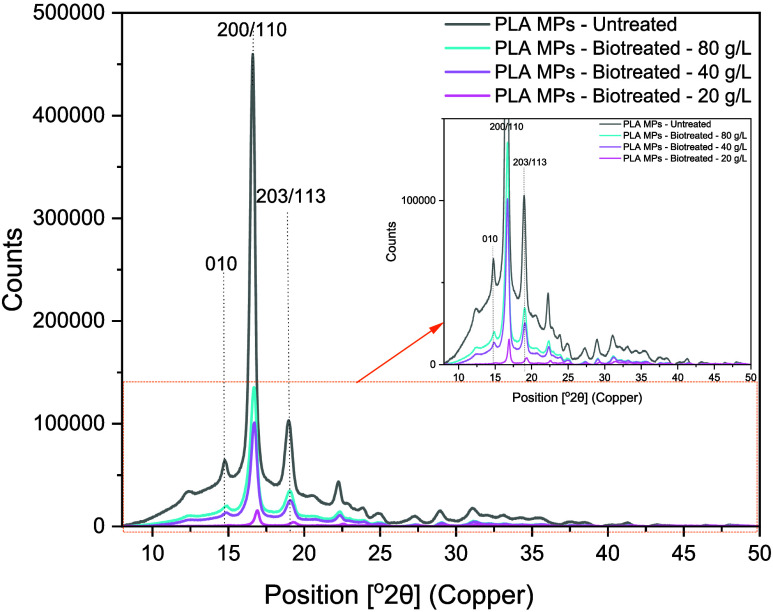
XRD diffractograms of untreated and biotreated PLA MPs with *P. ostreatus* after 30 days.

To further examine the biodegradation of the PLA MPs and to explore the production of lactic acid, the primary degradation product of PLA, along this process, analysis of the growth medium was performed by HPLC-RID. In all experiments (including the control), no detection of lactic acid was observed during the biotreatment, most probably due to its consumption by the fungus. To validate this theory, new sets of experiments were conducted with varying lactic acid concentrations (from about 140 to 32,600 mg/L), keeping the same nitrogen content in the medium and growth conditions. According to the results presented in Figure 5, it is apparent that the fungus *P. ostreatus* is capable of consuming lactic acid even if it is found at high concentrations in the growth medium. The removal efficiency reaches about 70% for the lowest initial concentration of lactic acid (∼140 mg/L), while it decreases as the initial concentration of lactic acid increases. The fungal mass concentrations achieved were 0.304 ± 0.021, 0.563 ± 0.124, 1.316 ± 0.025, and 1.348 ± 0.376 g/L for experiments with initial lactic acid concentrations of about 140, 640, 4,570, and 32,600 mg/L, respectively. To the best of authors’ knowledge, this is the first study that demonstrates the consumption of lactic acid as the primary carbon source by white-rot fungi. To conclude, herein, we demonstrated that the edible fungus *P. ostreatus* can be used for the degradation of PLA materials since it recognizes them as a carbon source for its growth. The findings highlight the metabolic capability of white-rot fungi to degrade PLA materials and their monomers, underscoring their important ecological role in the bioremediation of biopolymers in the environment.

**Figure 5:**
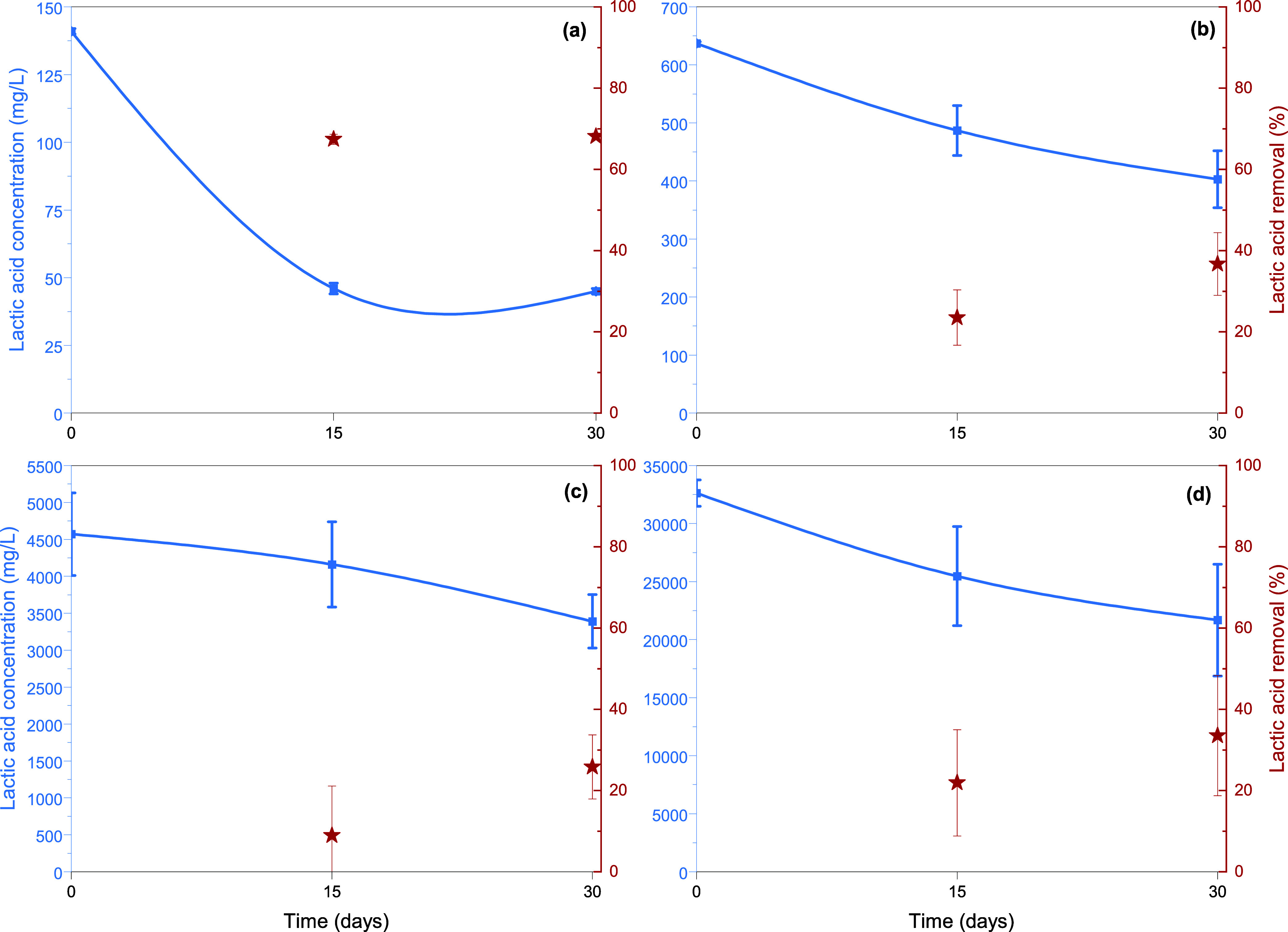
Lactic acid consumption (left blue axis) and removal (%) (right red axis) by the fungus *P. ostreatus* after 15 and 30 days of cultivation in a growth medium for various initial lactic acid concentrations of about (a) 140, (b) 640, (c) 4,570, and (d) 32,600 mg/L.

## 4 Conclusions

This study demonstrated the ability of *P. ostreatus* to degrade PLA microplastics under ambient conditions, revealing a mechanistic link between the fungal enzymatic activity and polymer chain scission. The degradation process was primarily attributed to the action of laccase, whose production increased proportionally with the PLA microplastic concentration, indicating that the fungus can recognize and utilize PLA as a carbon source. HPLC-RID analysis confirmed that *P. ostreatus* consumed lactic acid, the principal PLA degradation product, as a metabolic substrate. From a physical chemistry perspective, morphological and spectroscopic analyses provided direct evidence of bond cleavage and the oxidative modification of the polymer. SEM observations showed submicrometric surface perforations, while FTIR spectra revealed the formation of oxygen-containing functional groups (carbonyl, carboxyl, and hydroxyl), consistent with oxidative chain scission reactions. The observed increase in crystallinity indicates a preferential enzymatic attack on the amorphous regions of PLA, leading to a structural reorganization of the polymer matrix. These findings suggest that laccase can initiate radical-mediated oxidation and facilitate bond breakage within the polyester backbone without the need for elevated temperatures or pretreatment. Overall, edible white-rot fungi represent a promising, low-energy, and environmentally sustainable route for driving the oxidative depolymerization of PLA, offering new opportunities for integrating selective, enzyme-mediated end-of-life strategies into circular plastic management.

## Supplementary Material



## Notes

SEM images and FTIR spectra of PLA MPs (PDF)

## References

[1] European bioplastics . Bioplastic materials. https://www.european-bioplastics.org/bioplastics/materials/ (accessed Aug 23, 2024).

[2] Borrelle S.
B., Ringma J., Law K. L., Monnahan C. C., Lebreton L., Mcgivern A., Murphy E., Jambeck J., Leonard G. H., Hilleary M. A., Eriksen M., Possingham H. P., De Frond H., Gerber L. R., Polidoro B., Tahir A., Bernard M., Mallos N., Barnes M., Rochman C. M. (2020). Predicted
Growth in Plastic Waste Exceeds Efforts to Mitigate Plastic Pollution. Science.

[3] Gilani I. E., Sayadi S., Zouari N., Al-Ghouti M. A. (2023). Plastic
Waste Impact and Biotechnology: Exploring Polymer Degradation, Microbial
Role, and Sustainable Development Implications. Bioresour. Technol. Rep..

[4] Xi B., Wang B., Chen M., Lee X., Zhang X., Wang S., Yu Z., Wu P. (2022). Environmental Behaviors
and Degradation Methods of Microplastics in Different Environmental
Media. Chemosphere.

[5] Rosenboom J. G., Langer R., Traverso G. (2022). Bioplastics
for a Circular Economy. Nat. Rev. Mater..

[6] European bioplastics . Global production capacities of bioplastics 2028. https://www.european-bioplastics.org/news/multimedia-pictures-videos/#iLightbox[gallery_image_1]/2 (accessed Aug 23, 2024).

[7] Swetha T. A., Bora A., Mohanrasu K., Balaji P., Raja R., Ponnuchamy K., Muthusamy G., Arun A. (2023). A Comprehensive Review
on Polylactic Acid (PLA) – Synthesis, Processing and Application
in Food Packaging. Int. J. Biol. Macromol..

[8] Sahini M. G. (2023). Polylactic
Acid (PLA)-Based Materials: A Review on the Synthesis and Drug Delivery
Applications. Emergent Mater..

[9] Abu
Hajleh M. N., AL-Samydai A., Al-Dujaili E. A. S. (2020). Nano,
Micro Particulate and Cosmetic Delivery Systems of Polylactic Acid:
A Mini Review. J. Cosmet. Dermatol..

[10] Kulikowska D., Bernat K., Wojnowska-Baryła I., Pasieczna-Patkowska S., Jabłoński R. (2020). Composting as a Disposal Route of
Pla Materials: Kinetics of the Aerobic Biodegradation. Desalination Water Treat..

[11] Teixeira S., Eblagon K. M., Miranda F., R Pereira M. F., Figueiredo J. L. (2021). Towards Controlled Degradation of
Poly­(Lactic) Acid
in Technical Applications. C.

[12] Satti S. M., Shah A. A., Marsh T. L., Auras R. (2018). Biodegradation of Poly­(Lactic
Acid) in Soil Microcosms at Ambient Temperature: Evaluation of Natural
Attenuation, Bio-Augmentation and Bio-Stimulation. J. Polym. Environ..

[13] Hajighasemi M., Nocek B. P., Tchigvintsev A., Brown G., Flick R., Xu X., Cui H., Hai T., Joachimiak A., Golyshin P. N., Savchenko A., Edwards E. A., Yakunin A. F. (2016). Biochemical
and Structural Insights into Enzymatic Depolymerization of Polylactic
Acid and Other Polyesters by Microbial Carboxylesterases. Biomacromolecules.

[14] Karamanlioglu M., Houlden A., Robson G. D. (2014). Isolation
and Characterisation of
Fungal Communities Associated with Degradation and Growth on the Surface
of Poly­(Lactic) Acid (PLA) in Soil and Compost. Int. Biodeterior. Biodegrad..

[15] Beltrán F. R., de la Orden M. U., Lorenzo V., Pérez E., Cerrada M. L., Martínez
Urreaga J. (2016). Water-Induced Structural
Changes in Poly­(Lactic Acid) and PLLA-Clay Nanocomposites. Polymer.

[16] Lipsa R., Tudorachi N., Darie-Nita R. N., Oprică L., Vasile C., Chiriac A. (2016). Biodegradation of Poly­(Lactic
Acid)
and Some of Its Based Systems with Trichoderma Viride. Int. J. Biol. Macromol..

[17] Hino S., Kawasaki N., Yamano N., Nakamura T., Nakayama A. (2023). Effects of
Particle Size on Marine Biodegradation of Poly­(L-Lactic Acid) and
Poly­(ε-Caprolactone). Mater. Chem. Phys..

[18] Bertolacci L., Goldoni L., Zych A., Athanassiou A. (2022). Biocatalytic
Oxidation of Polyethylene by Agrocybe Aegerita Mycelium. Polym. Degrad. Stab..

[19] Andlar M., Rezić T., Marđetko N., Kracher D., Ludwig R., Šantek B. (2018). Lignocellulose
Degradation: An Overview of Fungi and
Fungal Enzymes Involved in Lignocellulose Degradation. Eng. Life Sci..

[20] Ramamurthy K., Thomas N. P., Gopi S., Sudhakaran G., Haridevamuthu B., Namasivayam K. R., Arockiaraj J. (2024). Is Laccase
Derived from Pleurotus Ostreatus Effective in Microplastic Degradation?
A Critical Review of Current Progress, Challenges, and Future Prospects. Int. J. Biol. Macromol..

[21] Shi Y., Zhu K., Dai Y., Zhang C., Jia H. (2020). Evolution and Stabilization
of Environmental Persistent Free Radicals during the Decomposition
of Lignin by Laccase. Chemosphere.

[22] Posada F., Malfreyt P., Gardette J.-L. (2001). Hydrogen
Abstraction from Poly­(Propylene)
and Poly­(Propylene Oxide) by Hydroxyl Radicals: A Computational Quantum
Semi-Empirical Study. Comput. Theor. Polym.
Sci..

[23] Bellettini M. B., Fiorda F. A., Maieves H. A., Teixeira G. L., Ávila S., Hornung P. S., Júnior A. M., Ribani R. H. (2019). Factors Affecting
Mushroom Pleurotus Spp. Saudi J. Biol. Sci..

[24] Economou C. N., Economou C. N., Philippoussis A. N., Diamantopoulou P. A. (2020). Spent Mushroom
Substrate for a Second Cultivation Cycle of Pleurotus Mushrooms and
Dephenolization of Agro-Industrial Wastewaters. FEMS Microbiol. Lett..

[25] Hidalgo J., Epelde L., Anza M., Becerril J. M., Garbisu C. (2023). Mycoremediation
with Agaricus Bisporus and Pleurotus Ostreatus Growth Substrates versus
Phytoremediation with Festuca Rubra and Brassica Sp. for the Recovery
of a Pb and γ-HCH Contaminated Soil. Chemosphere.

[26] Pozdnyakova N., Dubrovskaya E., Chernyshova M., Makarov O., Golubev S., Balandina S., Turkovskaya O. (2018). The Degradation of Three-Ringed Polycyclic
Aromatic Hydrocarbons by Wood-Inhabiting Fungus Pleurotus Ostreatus
and Soil-Inhabiting Fungus Agaricus Bisporus. Fungal Biol..

[27] Jin X., Yu X., Zhu G., Zheng Z., Feng F., Zhang Z. (2016). Conditions
Optimizing and Application of Laccase-Mediator System (LMS) for the
Laccase-Catalyzed Pesticide Degradation. Sci.
Rep..

[28] Gómez-Méndez L. D., Moreno-Bayona D. A., Poutou-Piñales R. A., Salcedo-Reyes J. C., Pedroza-Rodríguez A. M., Vargas A., Bogoya J. M. (2018). Biodeterioration
of Plasma Pretreated LDPE Sheets by Pleurotus Ostreatus. PLoS One.

[29] Maisto M., Zuzolo D., Tartaglia M., Prigioniero A., Ranauda M. A., Germinario C., Falzarano A., Castelvetro V., Sciarrillo R., Guarino C. (2024). Advances in Plastic
Mycoremediation: Focus on the Isoenzymes of the Lignin Degradation
Complex. Sci. Total Environ..

[30] da
Luz J. M. R., Paes S. A., Nunes M. D., da Silva M. de C. S., Kasuya M. C. M. (2013). Degradation of Oxo-Biodegradable Plastic by Pleurotus
Ostreatus. PLoS One.

[31] Da
Luz J. M. R., Paes S. A., Ribeiro K. V. G., Mendes I. R., Kasuya M. C. M. (2015). Degradation of Green Polyethylene by Pleurotus Ostreatus. PLoS One.

[32] Karimi-Avargani M., Bazooyar F., Biria D., Zamani A., Skrifvars M. (2020). The Special
Effect of the Aspergillus Flavus and Its Enzymes on Biological Degradation
of the Intact Polylactic Acid (PLA) and PLA-Jute Composite. Polym. Degrad. Stab..

[33] Penkhrue W., Khanongnuch C., Masaki K., Pathom-aree W., Punyodom W., Lumyong S. (2015). Isolation
and Screening of Biopolymer-Degrading
Microorganisms from Northern Thailand. World
J. Microbiol. Biotechnol..

[34] Zhang J., Gao D., Li Q., Zhao Y., Li L., Lin H., Bi Q., Zhao Y. (2020). Biodegradation of Polyethylene Microplastic Particles
by the Fungus Aspergillus Flavus from the Guts of Wax Moth Galleria
Mellonella. Sci. Total Environ..

[35] Sowmya H. V., Ramalingappa, Krishnappa M., Thippeswamy B. (2014). Degradation of Polyethylene by Trichoderma
Harzianum—SEM, FTIR, and NMR Analyses. Environ. Monit. Assess..

[36] Noor H., Satti S. M., Din S. ud., Farman M., Hasan F., Khan S., Badshah M., Shah A. A. (2020). Insight on Esterase
from Pseudomonas Aeruginosa Strain S3 That Depolymerize Poly­(Lactic
Acid) (PLA) at Ambient Temperature. Polym. Degrad.
Stab..

[37] Meza
Huaman S. M., Nicholson J. H., Brogan A. P. S. (2024). A General Route
to Retooling Hydrolytic Enzymes toward Plastic Degradation. Cell Rep. Phys. Sci..

[38] Spier, M. R. ; Peron-Schlosser, B. ; Paludo, L. C. ; Gallo-García, L. A. ; Zanette, C. M. Microalgae as Enzymes Biofactories. In Handbook of Microalgae-Based Processes and Products: Fundamentals and Advances in Energy, Food, Feed, Fertilizer, and Bioactive Compounds; Elsevier, 2020; pp 687–706.

[39] Economou C. N., Bertozzi S. M., Nardi M., Paul U. C., Fiorentini F., Ferrari G., Contardi M., Armirotti A., Fragouli D., Athanassiou A. (2025). Enhanced Biodegradation of Polylactic
Acid by Aspergillus Oryzae Lipase: Toward Sustainable Plastic End-of-Life
Solutions. Bioresour. Technol..

[40] Bacha A. U. R., Nabi I., Zaheer M., Jin W., Yang L. (2023). Biodegradation
of Macro- and Micro-Plastics in Environment: A Review on Mechanism,
Toxicity, and Future Perspectives. Sci. Total
Environ..

[41] Alfaro-Núñez A., Astorga D., Cáceres-Farías L., Bastidas L., Soto Villegas C., Macay K., Christensen J. H. (2021). Microplastic
Pollution in Seawater and Marine Organisms across the Tropical Eastern
Pacific and Galápagos. Sci. Rep..

[42] Economou C. N., Diamantopoulou P. A., Philippoussis A. N. (2017). Valorization of Spent Oyster Mushroom
Substrate and Laccase Recovery through Successive Solid State Cultivation
of Pleurotus, Ganoderma, and Lentinula Strains. Appl. Microbiol. Biotechnol..

[43] Philippoussis A., Diamantopoulou P., Papadopoulou K., Lakhtar H., Roussos S., Parissopoulos G., Papanikolaou S. (2011). Biomass, Laccase and Endoglucanase
Production by Lentinula Edodes during Solid State Fermentation of
Reed Grass, Bean Stalks and Wheat Straw Residues. World J. Microbiol. Biotechnol..

[44] Arora D. S., Gill P. K. (2001). Comparison of Two
Assay Procedures for Lignin Peroxidase. Enzyme
Microb. Technol..

[45] Silva M. L. C., Souza V. B. d., Santos V. d. S., Kamida H. M., Vasconcellos-Neto J.
R. T. d., Góes-Neto A., Bello Koblitz M. G. (2014). Production
of Manganese Peroxidase by Trametes Villosa on Unexpensive Substrate
and Its Application in the Removal of Lignin from Agricultural Wastes. Adv. Biosci. Biotechnol..

[46] Kister, G. ; Cassanas, G. ; Vert, M. Effects of Morphology, Conformation and Configuration on the IR and Raman Spectra of Various Poly­(Lactic Acid)­s; 1998 39.

[47] Oliveira M., Santos E., Araújo A., Fechine G. J. M., Machado A. V., Botelho G. (2016). The Role of Shear and
Stabilizer on PLA Degradation. Polym. Test.

[48] Odigbo C., Adenipekun C., Oladosu I., Ogunjobi A. (2023). Polyethylene Terephthalate
(PET) Biodegradation by Pleurotus Ostreatus and Pleurotus Pulmonarius. Environ. Monit. Assess.

[49] Paço A., Duarte K., da Costa J. P., Santos P. S. M., Pereira R., Pereira M. E., Freitas A. C., Duarte A. C., Rocha-Santos T. A. P. (2017). Biodegradation
of Polyethylene Microplastics by the Marine Fungus Zalerion Maritimum. Sci. Total Environ..

[50] Morsi R., Bilal M., Iqbal H. M. N., Ashraf S. S. (2020). Laccases and Peroxidases:
The Smart, Greener and Futuristic Biocatalytic Tools to Mitigate Recalcitrant
Emerging Pollutants. Sci. Total Environ..

[51] Dhakar K., Pandey A. (2013). Laccase Production
from a Temperature and PH Tolerant
Fungal Strain of Trametes Hirsuta (MTCC 11397). Enzyme Res..

[52] Majhy B., Priyadarshini P., Sen A. K. (2021). Effect of Surface Energy and Roughness
on Cell Adhesion and Growth-Facile Surface Modification for Enhanced
Cell Culture. RSC Adv..

[53] Zareidoost A., Yousefpour M., Ghasemi B., Amanzadeh A. (2012). The Relationship
of Surface Roughness and Cell Response of Chemical Surface Modification
of Titanium. J. Mater. Sci. Mater. Med..

[54] Wang D., Zeng N., Li C., Li Z., Zhang N., Li B. (2024). Fungal Biofilm Formation and Its
Regulatory Mechanism. Heliyon.

[55] Harding M. W., Marques L. L. R., Howard R. J., Olson M. E. (2009). Can Filamentous
Fungi Form Biofilms?. Trends Microbiol..

[56] Wang F., Terry N., Xu L., Zhao L., Ding Z., Ma H. (2019). Fungal Laccase Production from Lignocellulosic Agricultural Wastes
by Solid-State Fermentation: A Review. Microorganisms.

[57] Knežević A., Milovanović I., Stajić M., Lončar N., Brčeski I., Vukojević J., Ćilerdžić J. (2013). Lignin Degradation
by Selected Fungal Species. Bioresour. Technol..

[58] Khatoon N., Sahar N. I., Ndu U., Ali N., Jamal A., Ahmed S., Ali M. I. (2017). High-Throughput
Production of Peroxidase
and Its Biodegradation Potential toward Polymeric Material. Int. J. Environ. Sci. Technol..

[59] Fernández-Fueyo E., Castanera R., Ruiz-Dueñas F.
J., López-Lucendo M. F., Ramírez L., Pisabarro A. G., Martínez A. T. (2014). Ligninolytic
Peroxidase Gene Expression by Pleurotus Ostreatus: Differential Regulation
in Lignocellulose Medium and Effect of Temperature and PH. Fungal Genet. Biol..

[60] Hock, O. G. ; Lum, H. W. ; De Qin, D. ; Kee, W. K. ; Shing, W. L. The Growth and Laccase Activity of Edible Mushrooms Involved in Plastics Degradation Toxicology 2019; Vol. 15.

[61] Krikorian V., Pochan D. J. (2005). Crystallization
Behavior of Poly­(L-Lactic Acid) Nanocomposites:
Nucleation and Growth Probed by Infrared Spectroscopy. Macromolecules.

[62] Cuadri A. A., Martín-Alfonso J. E. (2018). Thermal,
Thermo-Oxidative and Thermomechanical
Degradation of PLA: A Comparative Study Based on Rheological, Chemical
and Thermal Properties. Polym. Degrad. Stab..

[63] Monsoor M. A., Kalapathy U., Proctor A. (2001). Improved Method for Determination
of Pectin Degree of Esterification by Diffuse Reflectance Fourier
Transform Infrared Spectroscopy. J. Agric. Food
Chem..

[64] Painter P. C., Starsinic M., Squires E., Davis A. A. (1983). Short Communications
Concerning the 1600 Cm-l Region in the i.r. Spectrum of Coal. Fuel.

[65] Lee J. C., Moon J. H., Jeong J. H., Kim M. Y., Kim B. M., Choi M. C., Kim J. R., Ha C. S. (2016). Biodegradability
of Poly­(Lactic Acid) (PLA)/Lactic Acid (LA) Blends Using Anaerobic
Digester Sludge. Macromol. Res..

[66] De
Jong S. J., Arias E. R., Rijkers D. T. S., Van
Nostrum C. F., Kettenes-Van Den Bosch J. J., Hennink W. E. (2001). New Insights
into the Hydrolytic Degradation of Poly­(Lactic Acid): Participation
of the Alcohol Terminus. Polymer.

[67] Gupta B., Revagade N., Hilborn J. (2007). Poly­(Lactic Acid) Fiber: An Overview. Prog. Polym. Sci..

