# Supplementary material for: Pleurotus ostreatus-Mediated Bioremediation of Polylactic Acid Microplastics: Unveiling a Sustainable Solution
Source: ACS Omega. 2025 Dec 5;10(49):60945–54. doi: 10.1021/acsomega.5c09999 (PMC12713503; doi:10.1021/acsomega.5c09999)
Supplement: Supplementary file 1 [file ao5c09999_si_001.pdf]

Supporting Information

Pleurotus ostreatus-Mediated Bioremediation of Polylactic Acid Microplastics: Unveiling a Sustainable Solution

Christina N. Economou[1*], Sine Mandrup Bertozzi[2], Giorgia Ferrari[1], Andrea Armirotti[2], Despina Fragouli[1], Athanassia Athanassiou[1*]

[1] Smart Materials, Istituto Italiano di Tecnologia (IIT), Via Morego 30, Genova 16163, Italy

[2] Analytical Chemistry Facility, Istituto Italiano di Tecnologia (IIT), Via Morego 30, Genova 16163, Italy

[*] Corresponding authors

Email addresses: christina.oikonomou@iit.it (Christina N. Economou), athanassia.athanassiou@iit.it (Athanassia Athanassiou)

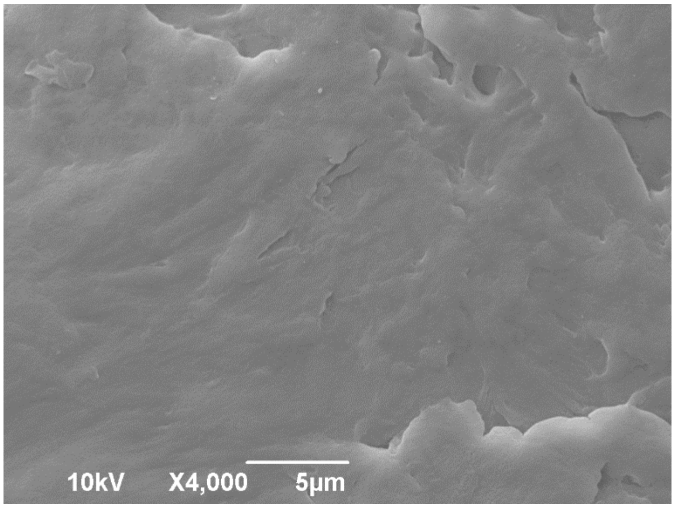

Figure 1S. SEM image of the surface of PLA MPs after *P. ostreatus* biotreatment with PLA MPs concentration in the growth medium of 7 g/L.

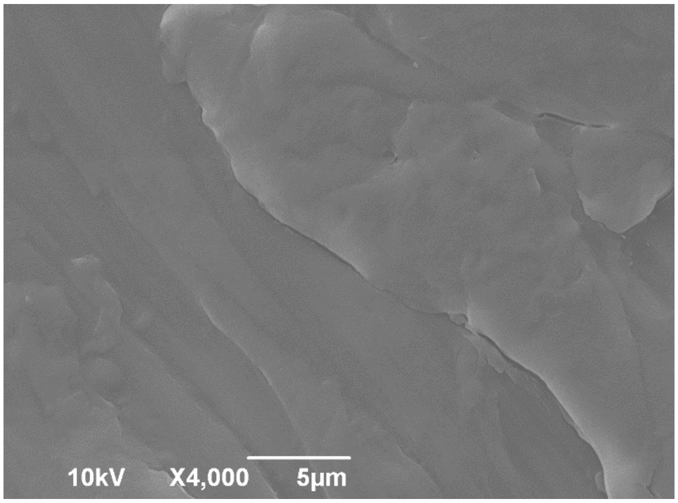

Figure 2S. SEM image of the surface of PLA MPs in control experiments (PLA MPs in the same growth medium without fungus) after 30 days.

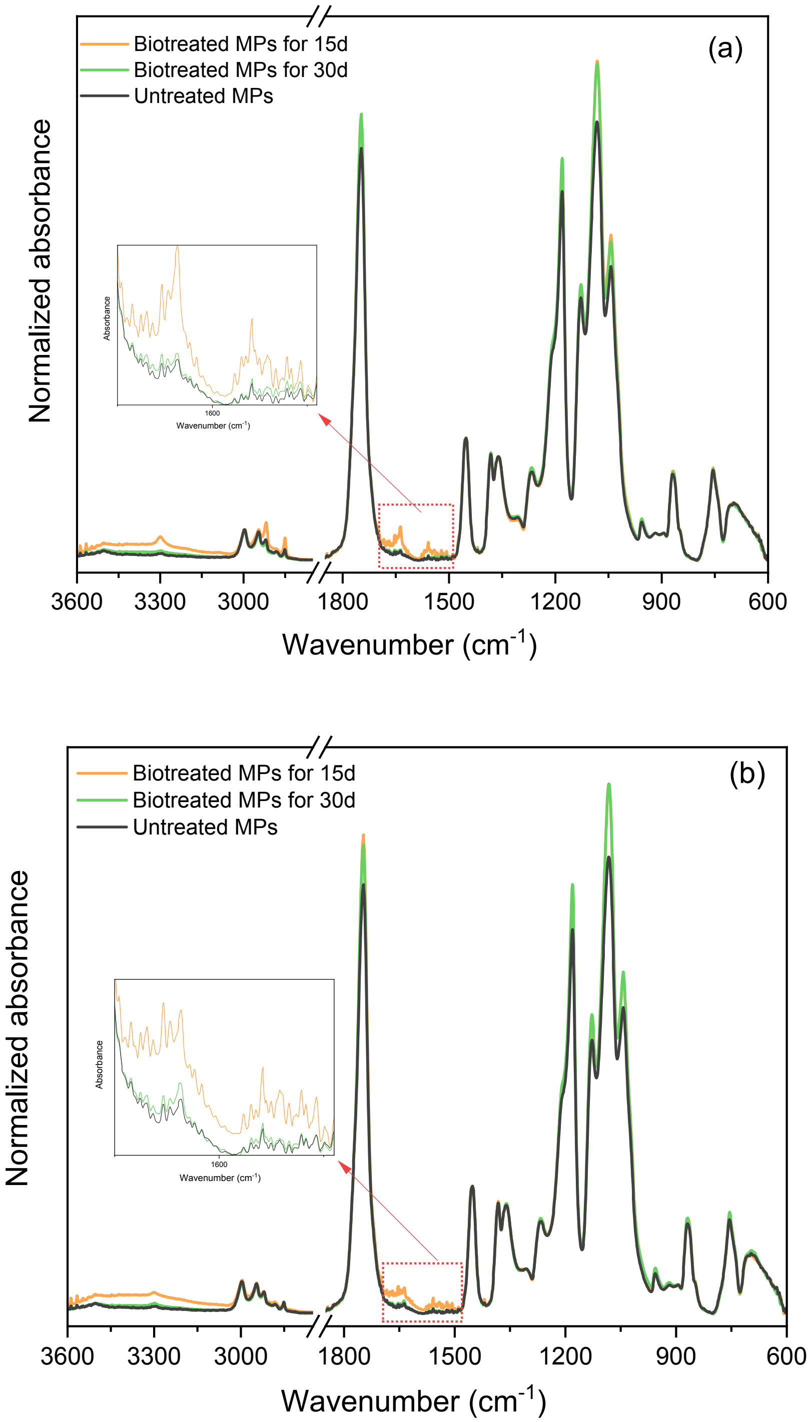

Figure 3S: Normalized ATR-FTIR spectra of PLA MPs of **(a)** 40 g/L and (**b**) 80 g/L in the growth medium after 15 and 30 days of *P. ostreatus* biotreatment in comparison with the untreated MPs spectrum.

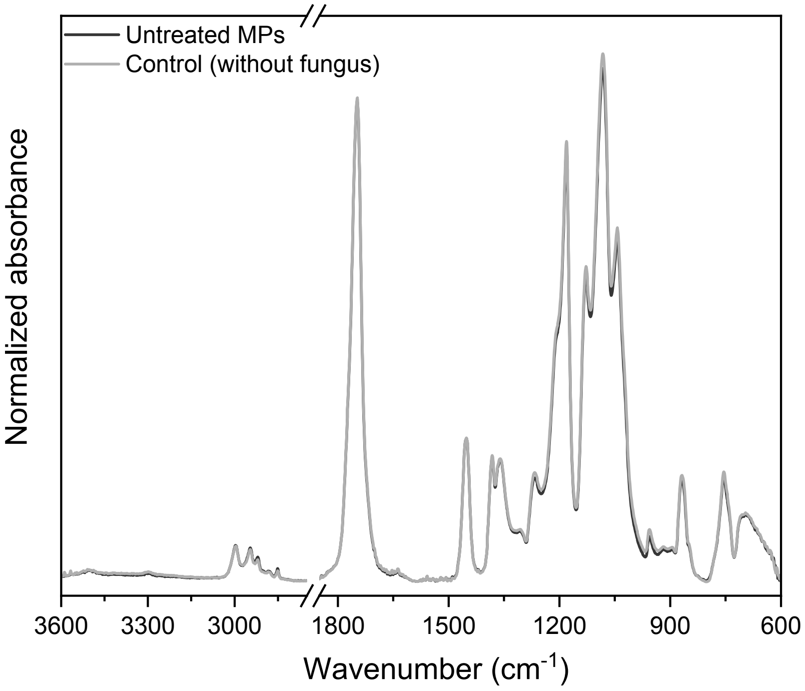

Figure 4S. Normalized ATR-FTIR spectra of untreated PLA MPs and control experiments (PLA MPs in the same growth medium without fungus) after 30 days.
